# Expression of immunoproteasome genes is regulated by cell-intrinsic and –extrinsic factors in human cancers

**DOI:** 10.1038/srep34019

**Published:** 2016-09-23

**Authors:** Alexandre Rouette, Assya Trofimov, David Haberl, Geneviève Boucher, Vincent-Philippe Lavallée, Giovanni D’Angelo, Josée Hébert, Guy Sauvageau, Sébastien Lemieux, Claude Perreault

**Affiliations:** 1 Institute for Research in Immunology and Cancer, Montreal, Quebec, Canada; 2 Department of Medicine, Université de Montréal, Montreal, Quebec, Canada; 3 Department of Computer Science and Operations Research, Université de Montréal, Montreal, Quebec, Canada; 4 Division of Hematology-Oncology, Maisonneuve-Rosemont Hospital, Montreal, Quebec, Canada; 5 Quebec Leukemia Cell Bank, Maisonneuve-Rosemont Hospital, Montreal, Quebec, Canada

## Abstract

Based on transcriptomic analyses of thousands of samples from The Cancer Genome Atlas, we report that expression of constitutive proteasome (CP) genes (*PSMB5, PSMB6, PSMB7*) and immunoproteasome (IP) genes (*PSMB8, PSMB9, PSMB10*) is increased in most cancer types. In breast cancer, expression of IP genes was determined by the abundance of tumor infiltrating lymphocytes and high expression of IP genes was associated with longer survival. In contrast, IP upregulation in acute myeloid leukemia (AML) was a cell-intrinsic feature that was not associated with longer survival. Expression of IP genes in AML was IFN-independent, correlated with the methylation status of IP genes, and was particularly high in AML with an M5 phenotype and/or *MLL* rearrangement. Notably, PSMB8 inhibition led to accumulation of polyubiquitinated proteins and cell death in IP^high^ but not IP^low^ AML cells. Co-clustering analysis revealed that genes correlated with IP subunits in non-M5 AMLs were primarily implicated in immune processes. However, in M5 AML, IP genes were primarily co-regulated with genes involved in cell metabolism and proliferation, mitochondrial activity and stress responses. We conclude that M5 AML cells can upregulate IP genes in a cell-intrinsic manner in order to resist cell stress.

All eukaryotes express constitutive proteasomes (CPs) that possess three catalytic subunits (PSMB5, PSMB6 and PSMB7). In addition to CPs, vertebrates also express immunoproteasomes (IPs), in which the catalytic β-subunits are replaced by IFN-γ–inducible homologues: PSMB8 for PSMB5, PSMB9 for PSMB6 and PSMB10 for PSMB7^1^. The first non-redundant role ascribed to IPs was their enhanced ability to generate MHC I-associated peptides^2^. However, recent work has revealed that IPs can be expressed by non-immune cell^3^,^4^ and that differential cleavage of transcription factors by CPs and IPs has pleiotropic effects on cell function^5^. Indeed, CPs and IPs differentially modulate the abundance of transcription factors that regulate signaling pathways with prominent roles in cell differentiation, inflammation and neoplastic transformation (e.g., NF-kB, IFNs, STATs and Wnt)^5^.

In cancer cells, genomic instability and oncogene addiction cause proteotoxic and oxidative stress^6^. Indeed, aneuploidy and variations in transcript levels produce imbalances in the stoichiometry of protein complexes and thereby lead to accumulation of misfolded proteins and formation of aggregates (proteotoxic stress)^7^,^8^,^9^. Moreover, oncogenic signaling and dysregulation of mitochondrial function generate reactive oxygen species which damage DNA and proteins (oxidative stress). Proteasomes are key players in stress response since they degrade damaged (misfolded or oxidized) proteins^10^,^11^,^12^. Accordingly, cancer cells are presumed to be unduly dependent on proteasomal function^13^. Besides, tumors are commonly infiltrated by IFN-γ-producing lymphocytes specific for neo-antigens^14^, and IFN-γ directly upregulates IP genes^1^. Hence, several factors could influence the abundance of proteasomes in neoplastic cells.

The goal of our work was therefore to determine whether CPs and IPs were differentially expressed in normal vs. neoplastic human cells and whether the two types of proteasomes played non-redundant roles in cancer cells. Here we report that overexpression of proteasomes is present in a wide variety of cancer types. Differential expression of CP genes had no impact on survival. However, IP upregulation in breast cancer showed a strong correlation with the abundance of interferon-producing tumor infiltrating lymphocytes and was associated with a good prognosis. In contrast, IP upregulation in AML was a cell-intrinsic feature that was not associated with improved survival. IP expression was particularly high in AML with an M5 phenotype according to the French-American-British (FAB) classification or in AML with an *MLL* rearrangement. IP expression in AML correlated with the methylation status of IP genes, and specific IP inhibition led to accumulation of polyubiquitinated proteins and cell death in IP^high^ but not IP^low^ AML cells. We conclude that expression of IP genes in human cancers is regulated by cancer cell-extrinsic (IFN-γ) and -intrinsic (cell stress) factors. Furthermore, our work identifies a functional vulnerability in IP^high^ AML cells because of an undue sensitivity to treatment with an IP-specific inhibitor.

## Results

### Genes encoding proteasome catalytic subunits are overexpressed in several cancer types

**Figure 1:**
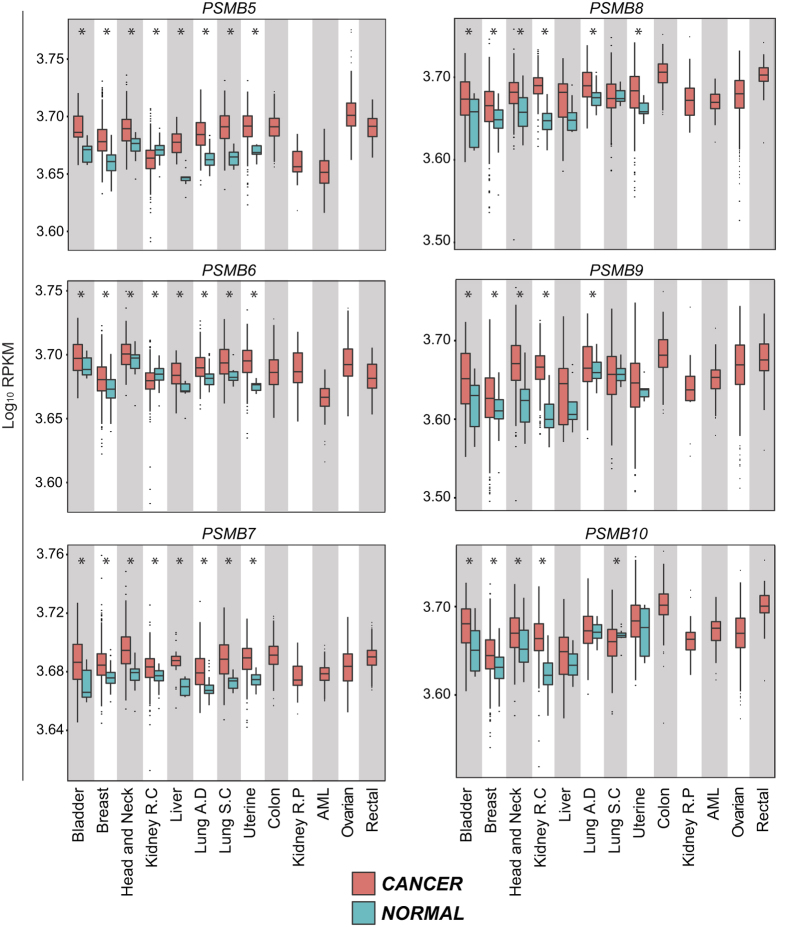
Genes encoding proteasome catalytic subunits are overexpressed in several cancer types. Boxplots of log_10_ [1000 × RPKM + 1] values for genes encoding proteasome catalytic subunits were drawn for the indicated cancer types. CP genes (on the left) are *PSMB5, PSMB6* and *PSMB7*, whereas IP genes (on the right) are *PSMB8, PSMB9 and PSMB10*. Red boxplots represent cancer samples and blue boxplots represent normal samples (when data are available). Differences in mean values between groups were determined by two-tailed unpaired Student’s t-tests. *Indicates *p* < 0.05. R.C: Renal Cell; R.P: Renal Papillary; AML: Acute Myeloid Leukemia; A.D: Adenocarcinoma; S.C: Squamous Cell.

In order to evaluate the expression of proteasome catalytic subunits in cancer, we first downloaded RNA-Seq data from TCGA, along with clinical metadata, from the Cancer Genomics Hub (see Methods). The initial analysis covered primary samples from thirteen tumor types from eleven different tissues, with normal tissue controls available for eight cancer types (Fig. 1). We analyzed the expression of the three CP- and the three IP-specific catalytic subunits. For the eight cancer types with available normal tissue controls, we found that a mean of five (out of six) proteasome catalytic subunits were slightly, but significantly, overexpressed in cancer samples (range 3–6) relative to normal tissue (Fig. 1). We conclude that proteasome upregulation is a general feature of cancer tissues.

### High expression of IP genes is associated with improved survival in breast cancer

**Figure 2:**
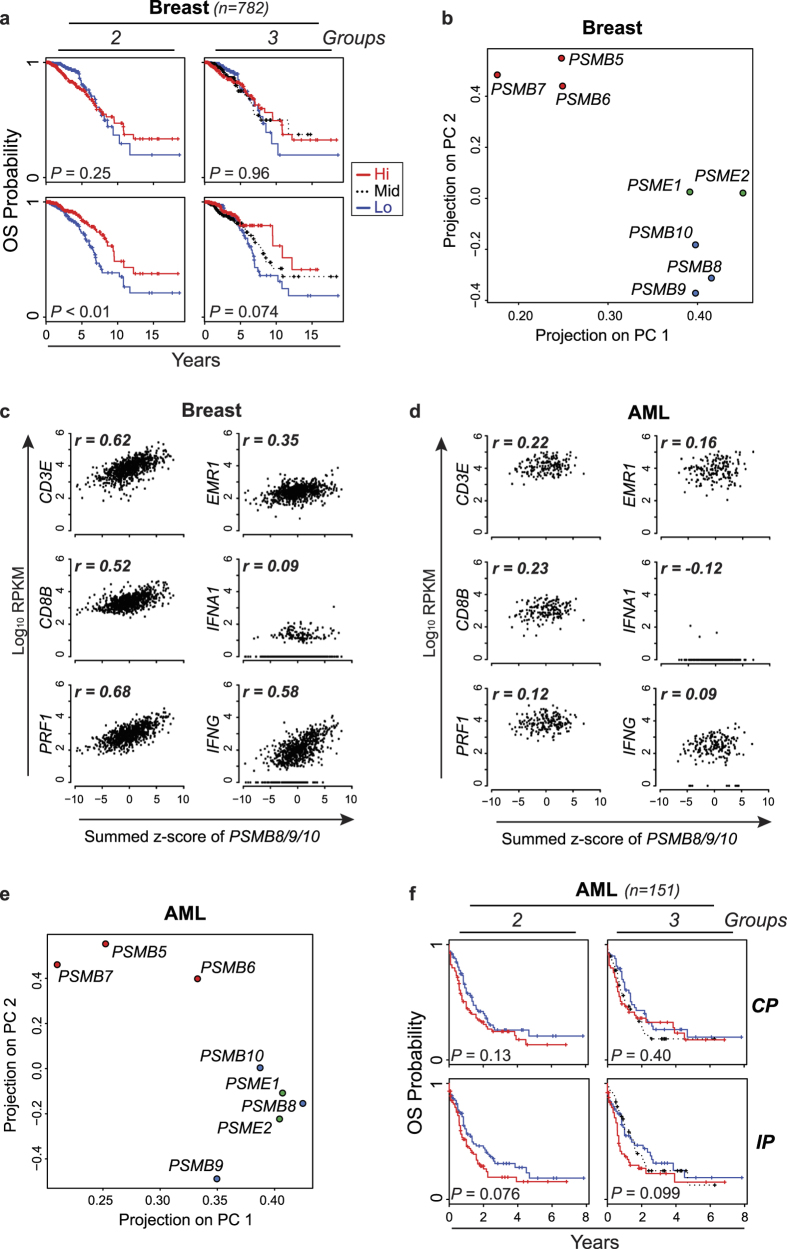
Expression of IP subunits is cell-autonomous in AML. (**a**) Kaplan-Meier plots of overall survival (OS) for CP^high^ vs. CP^low^ patients or IP^high^ vs. IP^low^ patients with breast cancer. The log-rank test was used to calculate *p*-values. (**b**) Principal component analysis was performed on log10 RPKM values for genes encoding CP, IP and regulatory cap subunits (PSME1 and PSME2) in breast cancer. Plots represent the projections on the first and second principal components (PC). (**c,d**) For AML and breast cancer samples from TCGA, the summed z-scores of *PSMB8/9/10* were plotted against log10 RPKM values of the indicated genes and Pearson’s correlation coefficient (***r***) was calculated. (**e**) Principal component analysis was performed for AML samples as described above. (**f**) Kaplan-Meier plots of overall survival (OS) for CP^high^ vs. CP^low^ patients or IP^high^ vs. IP^low^ patients in non-M3 AML samples. The log-rank test was used to calculate *p*-values.

We then sought to determine whether expression of CP- or IP-encoding genes correlated with survival in patients with various cancer types. For each patient in the TCGA cancer cohorts, expression of CP- or IP-encoding genes was transformed in z-score and summed. Based on this score, patient cohorts were separated in two or three groups of similar size (see Methods). This allowed us to evaluate the survival of patients with low or high expression of proteasome genes in their tumor sample. For most cancer types, expression of CP and IP genes showed no correlation with survival (Supplementary Fig. S1). However, IP gene expression did correlate with survival in breast cancer, as IP^high^ status was associated with a decreased risk of death (hazard ratio = 0.53 for 2 groups-Fig. 2a and Table 1). Indeed, survival at ten years was 61.9% ± 11.7% for patients whose IP gene expression ranked in the top third of the cohort (*IP*^*high*^) relative to 36.1% ± 8.0% for those in the bottom third (*IP*^*low*^) (Fig. 2a). Furthermore, expression of individual IP genes *PSMB8* and *PSMB10* was associated with a decreased risk of death (Supplementary Table S1). However, expression of CP genes did not correlate with survival in breast cancer: (i) high global expression of CP genes was not associated better prognosis when the cohort was separated in two or three groups (Fig. 2a), and (ii) no individual CP gene was associated with prolonged survival (Supplementary Table S1).

### IP subunits are co-expressed in breast cancer samples

In normal cells, assembly of IPs is cooperative: the three catalytic subunits (PSMB8, 9 and 10) interact with each other to favor their common incorporation in homogenous IPs^15^. However, intermediate proteasomes, containing CP and IP subunits, can be assembled and display some unusual proteolytic cleavage preferences^16^. To assess whether CP and IP catalytic subunits were co-expressed in breast cancer samples, we performed a principal component analysis (PCA) on gene expression data for CP- and IP-encoding genes and regulatory subunits PA28α and PA28β (encoded by *PSME1* and *PSME2*). PCA enriches for differences and variations by finding a rotation of the input data matrix that maximises the data variations in the first few dimensions. We found that, IP catalytic subunits clustered together with *PSME1* and *PSME2,* apart from the CP subunits (Fig. 2b). These results suggest that, like what is found in normal cells^3^, expression of IP subunits occurs in a coordinated manner in breast cancer cells.

### IP expression is cell-autonomous in AML but in not breast cancer

Expression of IPs can be upregulated by cell autonomous signaling or via paracrine secretion of IFN-γ by surrounding NK cells and CD8 T lymphocytes^17^. This is particularly relevant in tumors where CD8 + tumor-infiltrating lymphocytes (TILs) secrete copious amounts of IFN-γ^18^,^19^. We therefore asked whether IP expression correlated with the abundance of transcripts reflecting infiltration by CD8 TILs (*CD3E, CD8, PRF1*), macrophages (*EMR1*) and IFN secretion (*IFNA1, IFNG*). Based on RNA-Seq data from TCGA, expression of IP genes showed a strong correlation with expression of *IFNG* and T-cell genes in breast cancer (Fig. 2c). Since infiltration by CD8 TILs is associated with a good prognosis in many cancer types (including breast cancer)^20^, we surmise that the IP^high^ status in breast cancer is a marker of TIL infiltration and thereby correlates with prolonged survival (Fig. 2a). IP expression also correlated with the expression of *CD3E, CD8* and *PRF1* in colon cancer, another form of solid tumor infiltrated by TILs^21^ (data not shown).

Because infiltration by TILs has not been reported in hematologic malignancies such as AML, we then studied how IP expression was regulated in AML. We found that expression of IP subunits was coordinated in AML, but showed no significant correlation with infiltration by TILs nor with abundance of IFN transcripts (Fig. 2d–e). Furthermore, we observed a trend toward better prognosis in patients with IP^low^ relative to IP^high^ AML (Fig. 2f, Table 1 and Supplementary Table S1), but this trend did not reach statistical significance (*p* = 0.07 taking into consideration all AML subtypes except for acute promyelocytic leukemia–see Methods). Studies on additional patient cohorts will therefore be necessary in order to determine whether IP expression is a prognostic marker in AML. Nonetheless, these data reveal a clear dichotomy in IP regulation between breast cancer and AML, and beg the question: what is the nature of the cell autonomous (TIL-independent) signals that regulate IP levels in AML?

### IP subunits are highly expressed in myeloid and lymphoid cancer cell lines

We reasoned that if IP upregulation is cell-intrinsic in AML but TIL-dependent in breast cancer, we should detect higher IP levels in AML cell lines than in breast cancer cell lines (since cell lines contain no TILs). The transcriptional portrait of 675 human cancer cell lines was recently reported^22^. When we interrogated this resource, we found that, as predicted, IP genes were expressed at higher levels in myeloid leukemias (n = 21) than in breast cancer cell lines (n = 70) (Supplementary Fig. S2). When we analyzed other types of hematopoietic cancer cell lines, we found high expression of IP genes in lymphoid leukemia and lymphoma cell lines (Supplementary Fig. S2). Two points can be made from these data. First, they support the concept that high expression of IP genes is an intrinsic feature of AML but not breast cancer. Second, they suggest that overexpression of IP genes may be found not only in AML but also in other types of hematolymphoid malignancies.

### IP expression is upregulated in AML with an M5 phenotype or MLL rearrangement

**Figure 3:**
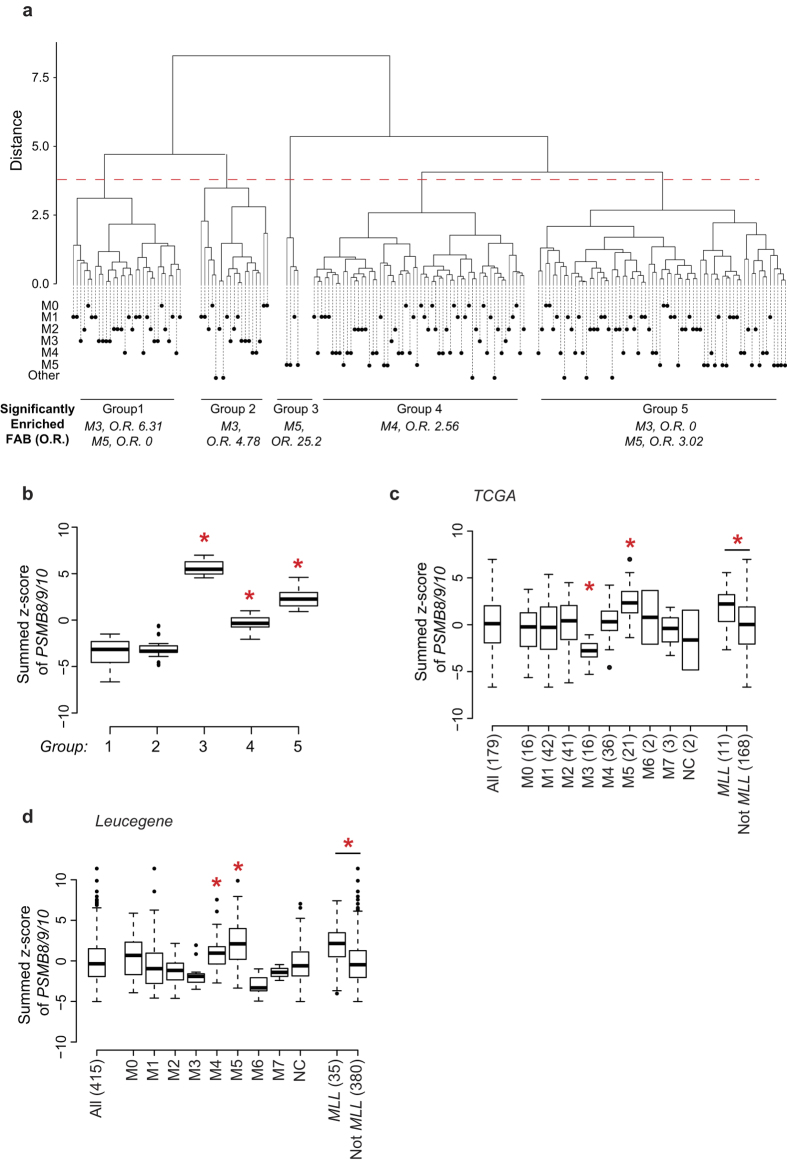
IP expression is upregulated in AML with an M5 phenotype or *MLL* rearrangements. (**a**) AML samples from TCGA were subjected to hierarchical clustering based on the expression of IP-encoding genes and separated into 5 clusters. Leafs of the dendrogram are annotated with circles that represent individual samples and their FAB subgroup. Enrichment in specific FAB classes within groups was determined by Fisher’s exact test and overall enrichment (O.R.) is shown. (**b**) Summed z-score of *PSMB8/9/10* in groups of patients separated by hierarchical clustering in (**a**). (**c,d**) Summed z-score of *PSMB8/9/10* expression in primary AML samples from (**c**) TCGA or (**d**) Leucegene cohorts were analyzed according to FAB classification and presence of *MLL* rearrangement. Differences between means of FAB groups were determined by one-way analysis of variance (ANOVA) followed by Tukey’s post-hoc test, and difference between means as a function of MLL status was determined by Student’s t-test (*indicates *p* < 0.05). Numbers in parentheses indicate number of samples.

AML is a complex and heterogeneous disease, which can be divided into distinct classes based on cytogenetic and molecular profiles^23^. Hence, to gain insights into IP regulation and function in primary AML samples, we performed a hierarchical clustering analysis of all TCGA AML samples based solely on the expression of IP-encoding genes (see Methods). This analysis led to the identification of five clusters of AML patients (Fig. 3a). Then, enrichment analyses were performed on each cluster for known cytogenetic markers, major translocations or morphologic subtypes (FAB classification) (see Methods). Only enrichment in FAB categories are shown since they yielded significant enrichments: patients with M3 AMLs were found to be enriched in clusters 1 and 2, which express low levels of IP, while M5 AMLs were enriched in clusters 3 and 5, which express high levels of IP (Fig. 3a,b). Furthermore, targeted classification of samples according to FAB subtypes confirmed that M5 AMLs expressed higher levels of IP than other FAB classes whereas M3 AMLs expressed lower IP levels (Fig. 3c). We also confirmed the overexpression of IP genes in M5 AMLs using the Leucegene cohort (415 samples), an independent cohort of AML samples with RNA-Seq data (Fig. 3d)^24^,^25^. In AML, *MLL* rearrangements are frequently associated to the M4 and M5 morphologic subtypes, while M3 AML are caused by promyelocytic leukemia/retinoic acid receptor-α (PML-RARA) oncoproteins^26^,^27^. Accordingly, we found superior IP gene expression in AMLs with MLL fusions in both the TCGA and Leucegene cohorts (Fig. 3c,d). Overall, these data show that IP expression is particularly high in AML with an M5 phenotype and/or *MLL* rearrangement.

### IP expression is regulated by DNA methylation

**Figure 4:**
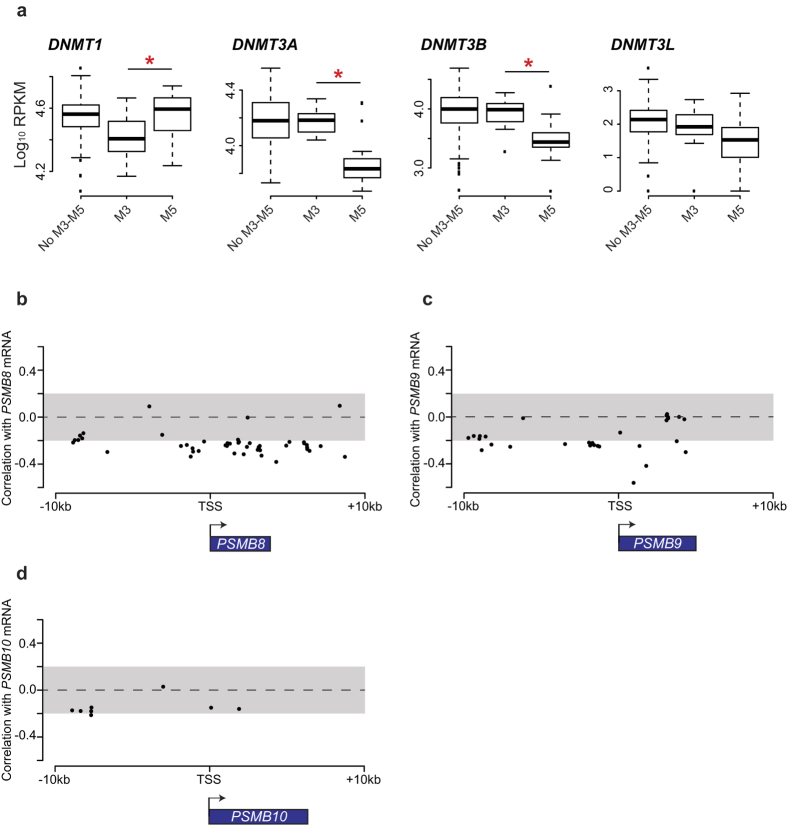
DNA methylation in primary AML samples. (**a**) Boxplots of log10 [1000 × RPKM + 1] values for the indicated genes in FAB M3 and M5 AMLs. Differences between means were determined by Student’s t-test. *Indicates *p* < 0.05. (**b–d**) Correlation coefficient between mRNA expression of (**b**) *PSMB8*, (**c**) *PSMB9* and (**d**) *PSMB10* and intensity of DNA methylation on cytosines located near coding sequences of *PSMB8/9/10* for all AML TCGA samples. Arrows indicates direction of transcription. TSS: Transcription Start Site.

**Figure 5:**
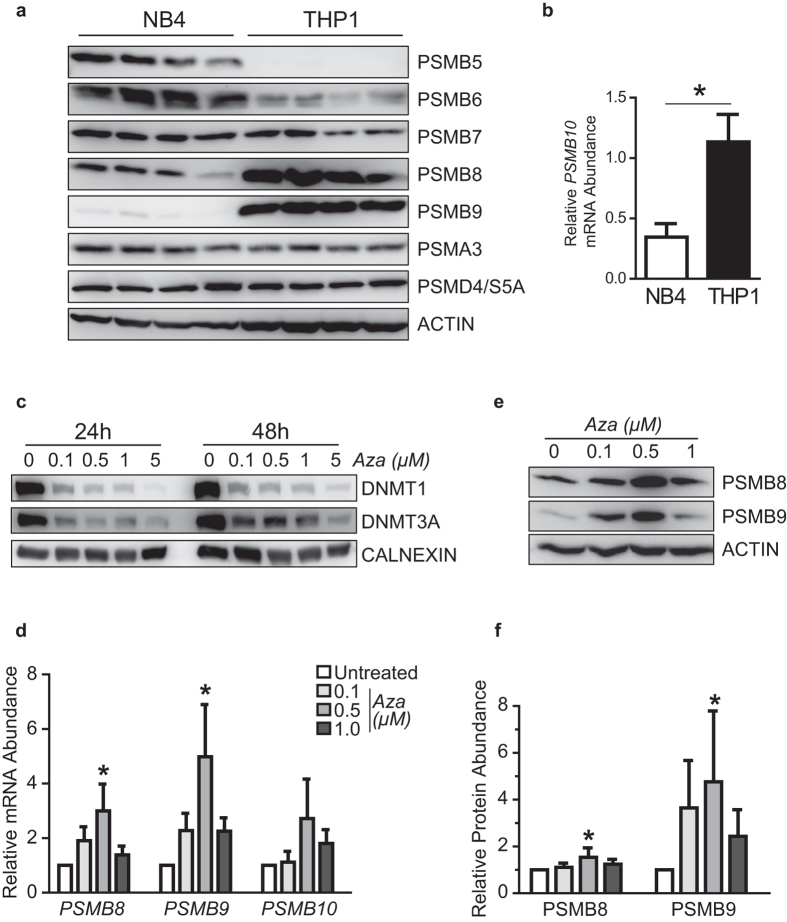
5-azacytidine treatment increases levels of *PSMB8* and *PSMB9* in NB4 cells. (**a**) Western blot analysis was performed to evaluate expression of proteasome subunits at the protein level in untreated THP1 and NB4 cells. β-actin serves as loading control. (**b**) Quantitative PCR analysis was performed on untreated THP1 and NB4 cells to determine total levels of *PSMB10* transcripts and data were normalized according to expression of *ACTB* and *TBP* (mean ± SD of four independent experiments). (**c–f**) NB4 cells were treated with the indicated concentrations of 5-azacytidine for (**c**) 24, 48 or (**e,f**) 72 hours followed by western blot analysis, or (**d**) for 48 hours followed by quantitative PCR analysis (mean ± SD of three independent experiments). Data were normalized according to expression of *ACTB* and *TBP* for quantitative PCR and β-actin or Calnexin served as loading control for western blot. Blots are representative of three independent experiments and were quantified using ImageJ software. Differences in means between groups were determined by one-way analysis of variance (ANOVA) followed by Dunnett’s post-hoc test. (*Indicates *p* < 0.05). Aza: 5-azacytidine.

Next, we sought to investigate the mechanisms responsible for the differential expression of IP subunits in AML, using M5 vs. M3 AMLs as a model for high and low IP-expressing cancer types. First, we analyzed whole-exome and whole-genome sequencing data available from TCGA for AML samples^28^. No recurrent mutation was present in the coding sequences of IP genes, or in upstream or downstream regulatory sequences (±9000 kb; data not shown), leaving epigenetics as a possible mechanism for dysregulation of IP expression in AML. Methylation of DNA is a stable epigenetic modification leading to transcriptional repression^29^. Interestingly, enzymes that control DNA methylation (*DNMT1* and *DNMT3A/B*) were differentially expressed between M3 and M5 AMLs (Fig. 4a). Furthermore, the intensity of DNA methylation on several cytosines located in the coding regions of *PSMB8* and *PSMB9* was inversely correlated to their gene expression (Fig. 4b–d). In order to investigate the role of DNA methylation in IP expression, we studied two cell lines: NB4 (M3, PML-RARA+) and THP1 (M5, *MLL-AF9* rearrangement). In accordance with our observations in primary AML samples, NB4 cells expressed low levels of IP genes, relative to the THP1 cells (Fig. 5a,b). Treatment with 5-azacytidine, an analog of cytidine inhibiting DNA methyltransferases^30^, reduced levels of DNMT1 and DNMT3A in NB4 cells after 24 and 48 hours (Fig. 5c). Notably, 5-azacytidine treatment increased the expression of PSMB8 and PSMB9, both at the mRNA (Fig. 5d) and protein level (Fig. 5e,f). We conclude that differential methylation of IP genes or their promoters can explain their contrasting expression levels in M3 vs. M5 AML. Nonetheless, further studies will be useful to determine the precise mechanisms responsible for the upregulation of IP genes by 5-azacytidine.

### IP expression correlates with distinct functional networks in M5 vs. non-M5 AML

**Figure 6:**
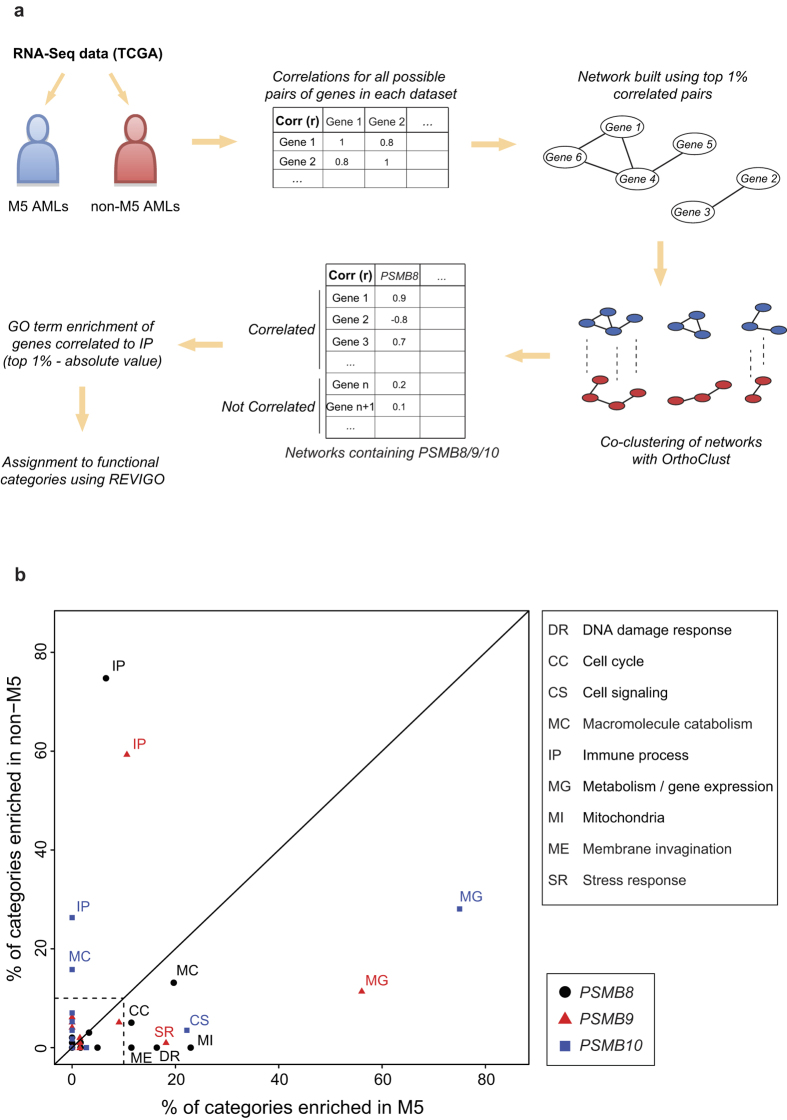
IP expression correlates with distinct functional networks in M5 vs. non-M5 AMLs. (**a**) Workflow for the analysis of functional networks in M5 vs. non-M5 AMLs. (**b**) Co-clustering was performed on aligned correlation networks derived from expression of all genes in M5 and non-M5 AMLs. GO term enrichment was performed on genes correlating with IP subunits within their respective cluster. Significantly enriched GO terms were split into general semantically similar process categories using Revigo. The plot represents the % of functional categories for each IP subunit, in M5 and non-M5 AMLs. Categories representing less than 10% are not shown.

Gene co-expression can yield systems-level insights into the function of genes and networks^31^. In order to investigate the role of IPs in AML subsets we split TCGA AMLs into two classes: M5 and non-M5. We then created correlation networks for each class and performed co-clustering using *OrthoClust* for both networks (Fig. 6a). We then retrieved the genes correlated to IP genes within each cluster and performed GO term enrichment on each gene set. Enriched GO terms were grouped into general categories and their distributions were compared (Fig. 6b and Supplementary Table S2). We found that genes correlated with IP subunits in non-M5 AMLs were primarily implicated in immune processes. In stark contrast, genes correlated with IP subunits in M5 AMLs were involved in metabolic and cell cycle processes, but not in immune processes. Moreover, genes correlated with PSMB8 and PSMB9 in M5 AMLs were enriched in processes linked to mitochondrial activity and stress responses, respectively. We conclude that while IP genes are mainly instrumental in immune processes in non-M5 AMLs, they are primarily connected to cell metabolism, proliferation and mitochondrial activity in M5 AMLs.

### THP1 cells are addicted to IPs

**Figure 7:**
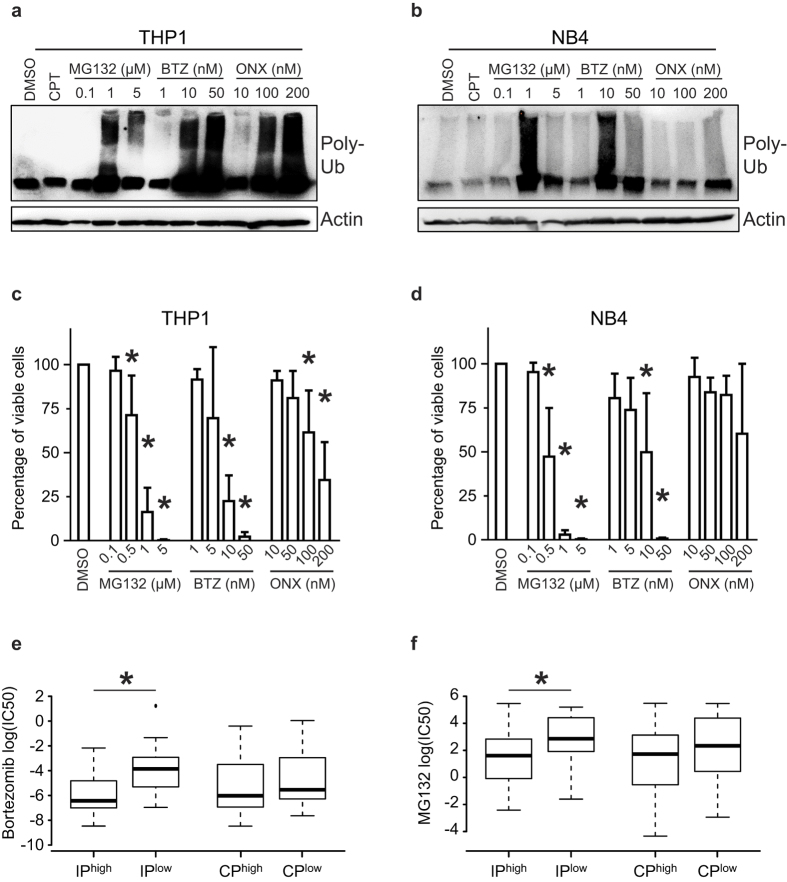
THP1 cells are addicted to IPs. (**a**) THP1 or (**b**) NB4 cells were treated for 24 hours with DMSO, 5 μM camptothecin (CPT), or with the indicated concentrations of MG132, Bortezomib (BTZ) or ONYX-0914 (ONX). Then, western blot analysis was performed to estimate total levels of polyubiquitinated proteins. β-actin served as loading control and blots are representative of three independent experiments. (**c**) THP1 or (**d**) NB4 cells were treated for 72 hours with DMSO or with the indicated concentrations of MG132, Bortezomib (BTZ) or ONYX-0914 (ONX), then cell viability was monitored using cell viability luminescence assay (mean ± SD of four independent experiments). Intensities were normalized as a percentage of viable cells relative to the DMSO control. Differences in means between groups were determined by one-way analysis of variance (ANOVA) followed by Dunnett’s post-hoc test. (*Indicates *p* < 0.05). (**e,f**) Cell lines from the Genomics of Drug Sensitivity in Cancer database (n = 309) were ranked according to their mean expression of IP or CP genes. Cell lines that ranked above the 90^th^ percentile and below the 10% percentile with regards to proteasome expression were used to create IP^high^/IP^low^ and CP^high^/CP^low^ groups. We compared these groups for their sensitivity to (**e**) Bortezomib and (**f**) MG132. Differences in log (IC50) between groups were determined by paired Mann-Whitney test (*indicates *p* < 0.05).

If IP overexpression is linked to vital cell processes specifically in M5 AMLs, then M5 AMLs should be overly sensitive to IP inhibition. To test this hypothesis, we treated THP1 cells (M5, *MLL-AF9* rearrangement) and NB4 cells (M3, PML-RARA+) with non-selective proteasome inhibitors (MG132 and Bortezomib) and with the PSMB8-specific inhibitor ONX-0914^32^. THP1 and NB4 cells showed high and low IP expression, respectively (Fig. 5a,b). MG132 and Bortezomib increased the amounts of polyubiquitinated proteins and decreased the viability of both NB4 and THP1 cells (Fig. 7a–d). However, the two cell lines showed divergent responses to ONX-0914. Indeed, ONX-0914 caused a massive accumulation of polyubiquitinated proteins and decreased the viability of THP1 cells, but had no effect on NB4 cells (Fig. 7a–d). These data show that, at least for the THP1 cell line, IP overexpression in AML cells correlates with susceptibility to a selective IP inhibitor.

### IP expression correlates with sensitivity to non-selective proteasome inhibitors

While inhibition of proteasome activity is effective for treatment of several cancer types^13^, there is limited knowledge about mechanisms of resistance to proteasome inhibitors^33^. To directly evaluate whether IP expression might regulate resistance to proteasome inhibition, we downloaded data from the Genomics of Drug Sensitivity in Cancer database, a public resource of drug responsiveness and gene expression data collected from a large panel of human cancer cell lines^34^. We ranked the 309 cell lines with available pharmacogenomic data according to their expression of IP or CP (irrespective of their cell lineage). Then top 10% and bottom 10% groups were used to compare their sensitivity to Bortezomib and MG132. Interestingly, IP^high^ cells were more sensitive to Bortezomib or MG132 treatment than IP^low^ cells, while no differences were observed between CP^high^ and CP^low^ cells (Fig. 7e,f). These analyses show that high IP expression is a marker of sensitivity to non-selective proteasome inhibitors.

## Discussion

The present work shows that expression of CP and IP genes is increased in most cancer types. Regulation of IP genes is of particular interest because it is regulated by both cell-intrinsic and –extrinsic factors in different types of cancer. We found that in breast cancer, upregulation of IP genes is a cancer cell-extrinsic process correlating with the presence of IFN-γ-secreting tumor-infiltrating lymphocytes. Hence, in accordance with the fact that lymphocyte infiltrates and IFN-γ secretion in solid tumors are favorable prognostic markers^14^,^35^,^36^, high expression of IP genes correlated with improved survival in patients with breast cancer. In contrast, in AML, upregulation of IP genes did not correlate with improved survival. Furthermore, levels of IP transcripts in AML were found to be IFN-independent and cell-intrinsic features associated with hypomethylation of IP genes. In AML, the three CP genes (PSMB5, 6 and 7) are co-expressed, as are the three IP genes (PSMB8, 9 and 10), but the CP and the IP trios are regulated independently (Fig. 2d). In line with this, the total amounts of proteasomes were similar in NB4 and THP1 cells (cf the non-catalytic subunits PSMA3 and PSMD4), but NB4 cells express mainly PSMB5, 6 and 7 CP units whereas THP1 contain mainly PSMB8, 9 and 10 IP units (Fig. 5a,b). This is consistent with evidence that IP gene upregulation leads to replacement of CPs with IPs rather than to the addition of IPs to CPs^37^. Replacement of CPs with IPs can have far reaching consequences. Indeed, these two types of proteasomes show differences in kinetics of substrate processing and in cleavage preferences that can lead to differential expression of thousands of genes^5^,^38^,^39^.

What can explain the cell-autonomous upregulation of IP genes in some cancers, and particularly in AML? One IFN-γ-independent factor was shown to preferentially induce transcription of IPs over CPs: oxidative stress. Indeed, IP upregulation has been reported in cells affected by several degenerative diseases linked to oxidative stress including amyotrophic lateral sclerosis (neurons), Duchenne muscular dystrophy (myocytes) and macular degeneration (retinal cells)^40^,^41^,^42^,^43^. Notably, relative to normal hematopoietic cells, AML cells have an increased reliance on oxidative phosphorylation and a higher mitochondrial mass, suffer from dysregulated mitochondrial biogenesis and metabolism, and are more susceptible to oxidative stress^44^,^45^. Consistent with this, we found that in M5 AML, the IP network was enriched in genes involved in metabolic processes, mitochondrial function and stress responses (Fig. 6). We therefore speculate that IP upregulation in AML cells may be driven by oxidative stress. In fact, from an evolutionary perspective, dealing with oxidative and other forms of cell stress may be the most conserved role of IPs. *PSMB8* and *PSMB9* orthologues have been found in invertebrates (who have no adaptive immune system), including the most basal branch of Metazoans—the placozoan *Trichoplax adhaerens*^46^. In invertebrates, the role of *PSMB8* and *PSMB9* orthologues is to help cells dealing with oxidative and proteotoxic stress. The notion that IPs are important for response to proteotoxic and oxidative stress is consistent with their expression in non-immune cells and their implication in functional processes such as cell differentiation and self-renewal^3^,^5^,^47^. Furthermore, we noted that cells expressing high levels of IP (but not CPs) were unduly sensitive to both selective IP inhibitors and unselective proteasome inhibition (Fig. 7). This supports the notion that cell-autonomous IP upregulation is driven by proteotoxic and oxidative stress in cancer cells.

Irrespective of the mechanisms causing IP upregulation in M5 AML cells, their susceptibility to a selective IP inhibitor (Fig. 7) identifies a functional vulnerability that warrants further studies. Unselective proteasome inhibitors have anti-myeloma and anti-AML activity^13^. However, since proteasomal activity is required for normal cell function and survival, unselective proteasome inhibitors cause substantial side effects and their therapeutic window is relatively narrow^13^. In contrast, transient inhibition of IPs has no effect on normal cells^48^. Hence, we propose that recently discovered IP-specific inhibitors^49^,^50^ could have substantial efficacy for treatment of IP^high^ AMLs (mostly AML with an M5 phenotype and/or MLL rearrangements), and perhaps other types of IP^high^ cancers, particularly those of hematopoietic origin (Supplementary Fig. S2). Systematic analyses using various IP-specific inhibitors on a large panel of hematopoietic cancer cell lines will be required in order to evaluate the potential clinical relevance of IP-specific inhibitors. The correlation that we found between IP gene methylation and expression could also be relevant to AML treatment regimens containing hypomethylating agents, which are being used particularly often in elderly subjects. Indeed, expression of DNMT3A is decreased in M5 AMLs (Fig. 4a), and mutations affecting this gene are known to be common and to have a negative impact on prognosis in AML^51^. Furthermore, we noted that treatment of the NB4 cell line with the hypomethylating drug 5-azacytidine increased IP expression (Fig. 5d–f). Hence, since our work suggests that IPs help AML cells to withstand oxidative stress, it might be advantageous to include IP-specific inhibitors to regimens containing hypomethylating agents.

## Methods

### Gene expression data

For normal and cancer tissues, RNA sequencing (RNA-Seq) datasets were downloaded from The Cancer Genome Atlas (TCGA) Data Portal Hub (https://tcga-data.nci.nih.gov/tcga/; BLCAv3.1.1, BRCAv3.1.2, COADv3.3.4, HNSCv3.1.4, KIRCv3.1.1, KIRPv3.1.1, LAMLv3.1.7, LIHCv3.1.0, LUADv3.1.2, LUSCv3.1.3, OVv3.1.5, READv3.1.2, and UCECv3.2.7). RPKM values from each patient were log-transformed (log_10_ [1000*RPKM + 1]) for normal/cancer comparison. For all other analyses, log-transformed expression of individual proteasome catalytic subunits (*PSMB5, PSMB6* and *PSMB7* for CP, and *PSMB8, PSMB9 and PSMB10* for IP) was transformed to z-score and summed to get global CP or IP z-scores. We defined z-scores as [gene X expression for a given sample–mean gene X expression of all samples]/standard deviation for gene X expression values in all samples]. Pearson’s correlation coefficients were calculated between global IP z-scores and log-transformed RPKM expression of indicated genes. For analysis human cancer cell lines, RNA-Seq datasets were obtained from the ArrayExpress database under accession number E-MTAB-2706 (http://www.ebi.ac.uk/arrayexpress/experiments/E-MTAB-2706/)^22^. Mean expression of IP genes (Gene IDs for *PSMB8*: 5696, *PSMB9*: 5698 and *PSMB10*: 5699) was calculated for specific cancer cell lineages according to Klijn *et al*.^22^. The IC50 values for Bortezomib and MG132 as well as gene expression data across 309 cancer cell lines were obtained from http://www.cancerrxgene.org/34. Cell lines were ranked according to their mean expression of IP or CP genes, then top 10% (“high”) and bottom 10% (“low”) groups were isolated and IC50 values of Bortezomib and MG132 were compared between both groups.

### Kaplan-Meier curves and survival analyses

For all cancer types, clinical datasets were downloaded from the TCGA Data Portal Hub and relevant information was extracted from clinical_patient_XXX.txt files. Columns labeled “vital_status” were used for patient status (expired or living) and columns labeled “days_to_death” and “days_to_last_followup” were used for survival analysis. Analyses were conducted using the R package *survival* (https://cran.r-project.org/web/packages/survival/index.html). Samples were divided in two or three equal groups based on global CP or IP z-scores. Cox proportional hazards models were used to estimate hazard ratios (high group/low group) and 95% confidence intervals. The log-rank test was used to calculate *p*-values corrected for three or more comparisons. Since they were not treated with cytarabine-based protocols protocols (like other AML sub-groups), patients with acute promyelocytic leukemia (n = 16) were not included in analyses of patient survival (Fig. 2 and Table 1).

### Analysis of DNA methylation

For AML, DNA methylation datasets were downloaded from the TCGA Data Portal Hub and methylation intensity (Beta-value) was retrieved for all sites (n = 485, 577). We kept CpG sites for which Beta-value was correlated >0.2 or <−0.2 with the log-transformed RPKM expression of either *PSMB8, PSMB9* or *PSMB10* (Pearson’s correlation) in all AML samples. CpG sites present <10 kb upstream or downstream of the transcription start site of *PSMB8, PSMB9 or PSMB10* were plotted against their level of correlation with expression of *PSMB8, PSMB9* or *PSMB10*.

### Cell culture

THP-1 and NB4 cell lines were obtained from Dr. Brian Wilhelm (IRIC, Université de Montréal, Canada) and maintained in RPMI-1640 media supplemented with 10% fetal bovine serum and 100 U/mL penicillin-streptomycin (Thermo Fisher Scientific, Waltham, MA). For cell viability assays, THP1 and NB4 cells were seeded at 5 × 10^4^ cells in 100 μl in 96-well plates and treated for 72 hours with MG132 (EMD millipore, Etobicoke, Canada), Bortezomib (New England Biolabs, Whitby, Canada) or ONX-0914 (Cayman Chemicals, Ann Harbor, MI). CellTiter-Glo Luminescent Viability reagent (Promega, Madison, WI) was added to the wells according to manufacturer’s instructions and signal was read using a plate reader. For inhibition of DNA methylation assays, NB4 cells were seeded at 5 × 10^5^ cells in 1 mL in 24-well plates and treated 48 or 72 hours with 5-azacytidine (Sigma-Aldrich, St-Louis, MO).

### RT-qPCR analyses

Total RNA was extracted from cells with TRIzol RNA reagent (Thermo Fisher Scientific) and retro-transcribed with the High-Capacity cDNA Reverse Transcription kit (Thermo Fisher Scientific). Quantitative PCRs were performed using Taqman technology with ViiA™ 7 Real-Time PCR system (Thermo Fisher Scientific) and results were analysed with the ViiA™ 7 software. Primer sequences were designed with the Universal Probe Library system (Roche Life Sciences, Madison, WI) as follows: PSMB8 (Fwd: accccgcgtgacactact, Rev: gggactggaagaattctgtgg, Probe #17), PSMB9 (Fwd: accaaccggggacttacc, Rev: tcaaacactcggttcaccac, Probe #86), PSMB10 (Fwd: ggttccagccgaacatga, Rev: gcccaggtcacccaagat, Probe #31).

### Western blot analyses

Cells were lysed in RIPA buffer (50 mmol/L Tris-HCl pH 7.4, 1% Nonidet P-40, 0.25% Na-deoxycholate, 150 mmol/L NaCl, 1 mmol/L EDTA) containing complete protease inhibitor mixture (Roche Life Sciences), 1 mmol/L Na_3_VO_4_ pH 9, 5 mmol/L NaF and 10mM NEM (Sigma-Aldrich). Samples were resolved by SDS-PAGE and immunoblotted with the following antibodies: anti-β1/PSMB6, anti-β5/PSMB5, anti-LMP2/PSMB10, anti-LMP7/PSMB8 (Abcam, Cambridge, UK); anti-β2/PSMB7, anti–poly-Ub (FK1 clone) (Enzo Life Sciences, Farmingdale, NY); anti-PSMA3, anti-S5A/PSMD4 (New England Biolabs); anti-β-actin (AC-15) (Sigma-Aldrich). After incubation with anti-mouse (BD Bioscience, Franklin Lakes, NJ) or anti-rabbit (New England Biolabs) horseradish peroxidase–conjugated secondary antibodies, chemiluminescent signal was detected using SuperSignal™ West Femto Detection Kit (Thermo Fisher Scientific) and an ImageQuant LAS-4000 imaging system (Fujifilm, GE Healthcare, Baie d’Urfe, QC, Canada).

### Hierarchical clustering

Using R statistical software, the Euclidean distance was calculated for all TCGA AML samples (n = 179), according to expression of *PSMB8, PSMB9, PSMB10*, followed by hierarchical clustering. The clustering results are shown in a dendrogram tree, built using the R package *ggdendro* (https://cran.r-project.org/web/packages/ggdendro/index.html). The tree was manually separated into 5 clusters and Fisher’s exact tests were performed to determine enrichment in FAB categories within each cluster.

### Co-clustering analysis

TCGA AML samples were split into M5 (n = 21) and non-M5 groups (n = 158). Correlation networks were built within each group based on log-transformed RPKM expression values. Network edges were drawn between two genes only when Pearson’s correlation absolute value between them was in the first centile. Networks were aligned using distribution similarity (Mann-Whitney-Wilcoxon test, p-value < 0.05) of a given gene in both groups, in order to enrich for gene similarities (same distribution and correlations) between groups during the co-clustering. Both networks and their alignment were fed to the computational framework *OrthoClust*^52^, which performed co-clustering of all genes.

### GO term enrichment

Within clusters containing IP-encoding genes (*PSMB8, PSMB9, PSMB10)*, all genes correlating with individual IP subunits were extracted, according to the top 1% threshold used previously. GO term enrichment was performed on these gene lists using an in-house written tool (https://github.com/TrofimovAssya/GOrichr) that implements a modified threshold Fisher’s exact test (Odds Ratio > 2) to avoid detection of slightly enriched terms in large groups. GO terms that passed these thresholds were grouped under general categories using REVIGO (http://revigo.irb.hr/)^53^ (Supplementary Table S2).

### Principal component analysis

The expression of each IP- and CP-encoding genes and of regulatory subunits *PSME1* and *PSME2* was normalized to z-score and put through a principal component analysis using R Statistical Software for breast cancer and AML samples. Samples were projected in two dimensions using the two components accounting for most of the sample variation.

## Additional Information

**How to cite this article**: Rouette, A. *et al*. Expression of immunoproteasome genes is regulated by cell-intrinsic and –extrinsic factors in human cancers. *Sci. Rep.* **6**, 34019; doi: 10.1038/srep34019 (2016).

## Supplementary Material

Supplementary Information

## Figures and Tables

**Table 1:** Correlation between risk of death and proteasome expression.

**Breast cancer**			
Category	# of groups	Hazard Ratio [95% Confidence Interval]	*p*-value
CP^high^ vs CP^low^	2	1.30 [0.83–2.02]	0.245
CP^high^ vs CP^low^	3	1.02 [0.78–1.33]	0.960
IP^high^ vs IP^low^	2	0.53 [0.35–0.82]	0.003
IP^high^ vs IP^low^	3	0.74 [0.56–0.97]	0.074
**AML**			
Category	# of groups	Hazard Ratio [95% Confidence Interval]	*p*-value
CP^high^ vs CP^low^	2	1.35 [0.91–1.99]	0.133
CP^high^ vs CP^low^	3	1.28 [0.79–2.08]	0.404
IP^high^ vs IP^low^	2	1.42 [0.96–2.11]	0.076
IP^high^ vs IP^low^	3	1.54 [0.95–2.51]	0.099

Patients were divided into two or three equal groups based on CP or IP z-score. Patients with M3 AML (n = 16) were not included in the analysis because they were not treated with the same chemotherapy regimen as other AML patients. Cox proportional hazards models were used to estimate hazard ratios (high group/low group) and 95% confidence intervals. The log-rank test was used to calculate *p*-values.
