# Supplementary material for: Expression of immunoproteasome genes is regulated by cell-intrinsic and –extrinsic factors in human cancers
Source: Sci Rep. 2016 Sep 23;6:34019. doi: 10.1038/srep34019 (PMC5034284; doi:10.1038/srep34019)
Supplement: Supplementary Information [file srep34019-s1.pdf]

Expression of immunoproteasome genes is regulated by cell-intrinsic and –extrinsic factors in human cancers

Alexandre Rouette[1,2,6], Assya Trofimov[1,2,3,6], David Haberl[1], Geneviève Boucher[1], Vincent-Philippe Lavallée[1,2,4,5], Giovanni D'Angelo[4,5], Josée Hébert[1,2,4,5], Guy Sauvageau[1,2,4,5], Sébastien Lemieux[1,3] and Claude Perreault[1,2,4,*]

[1]Institute for Research in Immunology and Cancer; [2]Department of Medicine and [3]Department of Computer Science and Operations Research, Université de Montréal; [4]Division of Hematology-Oncology and [5]Quebec Leukemia Cell Bank, Maisonneuve-Rosemont Hospital, Montreal, Quebec, Canada;

[6]These authors contributed equally to this work

[*]CORRESPONDENCE: Claude Perreault (claude.perreault@umontreal.ca), Institute for Research in Immunology and Cancer, P.O. Box 6128, Station Centre-Ville, Montreal, QC, Canada, H3C 3J7. Phone: 514-343-6126; Fax: 514-343-5839

This PDF includes:

Supplemental Table S1. Risk of death associated to expression of proteasome encoding genes.

Supplemental Table S2. Functional categories associated to genes specifically correlating with IP in M5 or non-M5 AMLs

Supplemental Figure S1. Correlation of CP or IP expression with survival outcome.

Supplemental Figure S2. IP expression in human cancer cell lines.

**Breast cancer**		
Gene Name	Hazard Ratio [95% Confidence Interval]	*p*-value
PSMB5	0.56 [0.15 – 2.12]	0.393
PSMB6	0.47 [0.11 – 2.06]	0.316
PSMB7	1.49 [0.29 – 7.65]	0.634
PSMB8	0.34 [0.18 – 0.63]	0.001
PSMB9	0.60 [0.34 – 1.04]	0.067
PSMB10	0.31 [0.14 – 0.66]	0.003
**AML**		
Gene Name	Hazard Ratio [95% Confidence Interval]	*p*-value
PSMB5	2.95 [0.82 – 10.7]	0.099
PSMB6	2.82 [0.66 – 12.1]	0.162
PSMB7	2.14 [0.19 – 24.4]	0.541
PSMB8	6.30 [1.56 – 25.6]	0.001
PSMB9	2.25 [0.88 – 5.73]	0.088
PSMB10	2.85 [0.91 – 8.93]	0.072

Supplementary Table S1. Risk of death associated to expression of proteasome encoding genes. Cox proportional hazards models were used to estimate hazard ratios, 95% confidence intervals and *p*-values associated with expression of proteasome-encoding genes as linear covariates.

Functional Category	M5 *PSMB8*	M5 *PSMB9*	M5 *PSMB10*	non-M5 *PSMB8*	non-M5 *PSMB9*	non-M5 *PSMB10*
Apoptotic process	3.3	1.5	0.0	3.0	2.1	0.0
Cell cycle	11.5	9.1	0.0	5.1	5.2	7.0
Death	1.6	1.5	0.0	0.0	0.0	0.0
DNA damage response	16.4	0.0	0.0	0.0	4.2	1.8
Immune process	6.6	10.6	0.0	74.8	59.4	26.3
Macromolecule catabolism	19.7	0.0	0.0	13.1	5.2	15.8
Membrane invagination	11.5	0.0	2.8	0.0	0.0	0.0
Mitochondria	23.0	0.0	0.0	0.0	0.0	3.5
Multi-organism process	1.6	1.5	0.0	1.0	1.0	5.3
Viral process	4.9	1.5	0.0	0.0	0.0	0.0
Metabolism and gene expression	0.0	56.1	75.0	1.0	11.5	28.1
Stress response	0.0	18.2	0.0	0.0	1.0	3.5
Cell signaling	0.0	0.0	22.2	2.0	6.3	3.5
Extracellular transport	0.0	0.0	0.0	0.0	4.2	0.0
Cellular component organisation	0.0	0.0	0.0	0.0	0.0	5.3

Supplementary Table S2. Functional categories associated to genes specifically correlating with IP in M5 or non-M5 AMLs. Enriched gene-ontology terms for genes specifically correlated to IP genes in M5 and non-M5 AMLs were assigned to functional categories using Revigo (see Figure 6 and Methods). Each functional category is depicted as a percentage of total categories for its column and plotted in Figure 6.

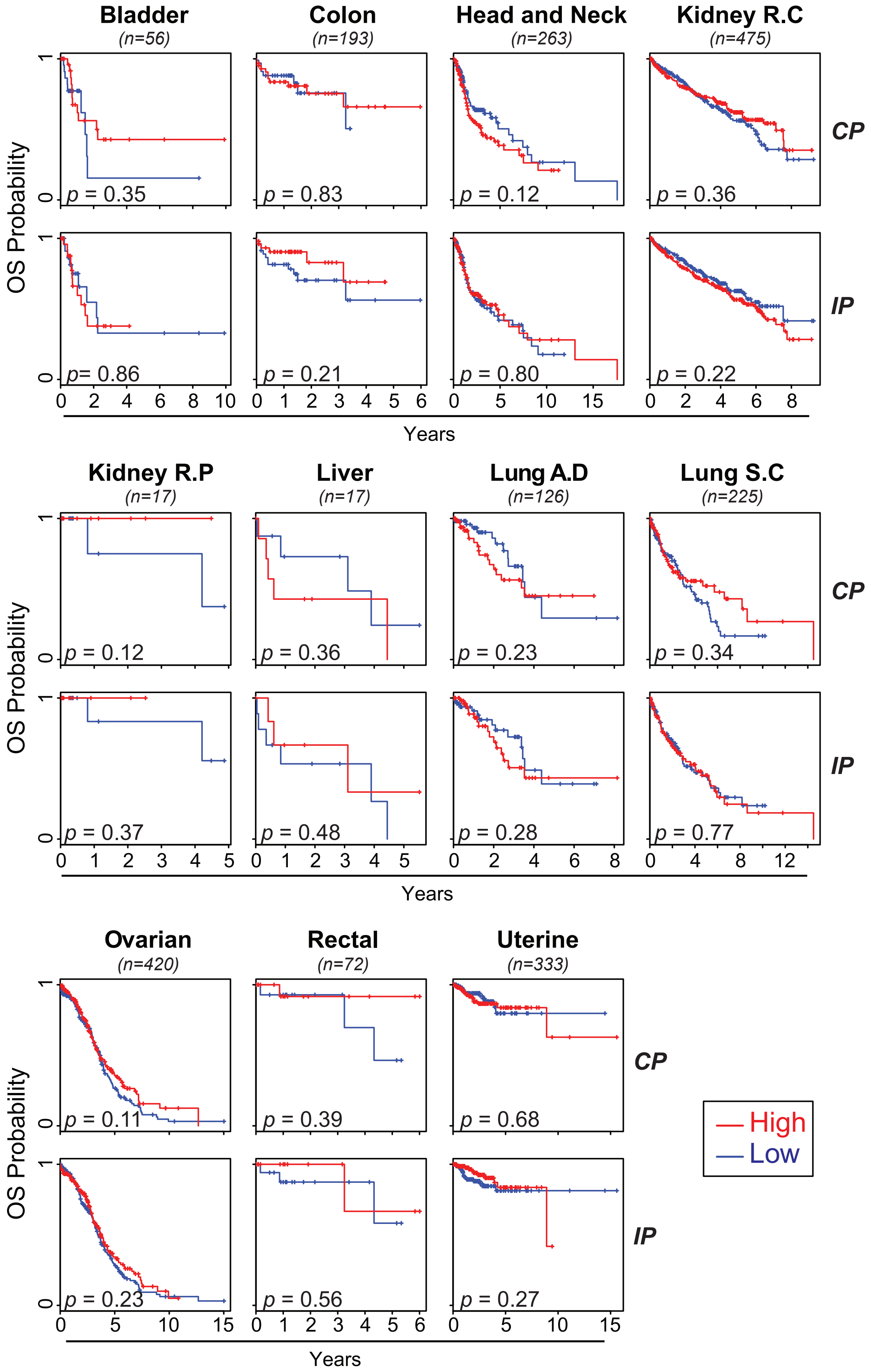

Supplementary Figure S1. Correlation of CP or IP expression with survival outcome. Patients were divided in two equal groups based on CP or IP z-score. Kaplan-Meier plots of overall survival (OS) for CP^{high}/CP^{low} patients, or IP^{high}/IP^{low} patients are shown for the indicated cancer type. *p*-values were calculated using the log-rank test. R.C: Renal Cell; R.P: Renal Papillary; A.D: Adenocarcinoma; S.C: Squamous Cell.

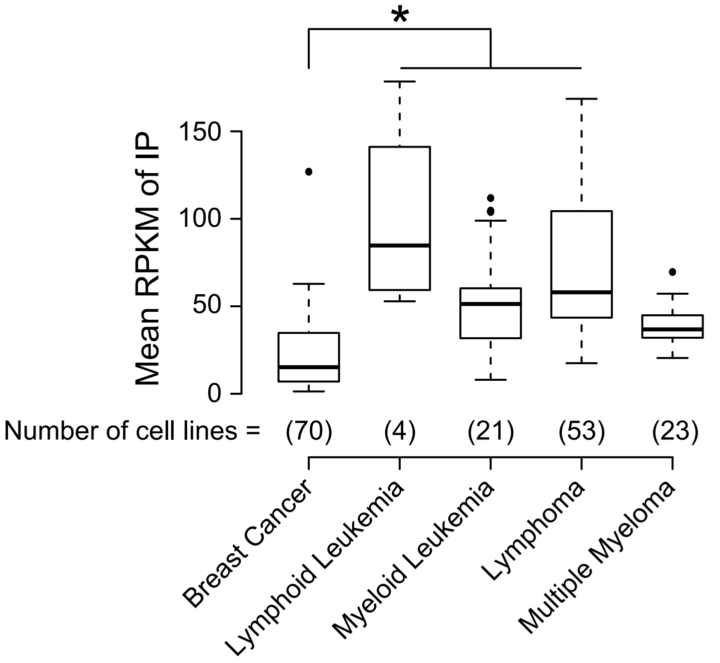

Supplementary Figure S2. IP expression in human cancer cell lines.
Boxplots of the mean RPKM of *PSMB8*, *PSMB9* and P*SMB10*. Numbers in parenthesis indicate the number of cell lines per disease type. Differences between means were determined by one-way analysis of variance (ANOVA) followed by Tukey's post-hoc test. * indicates $p < 0.01$.
